# A Novel Hybrid Dimension Reduction Technique for Undersized High Dimensional Gene Expression Data Sets Using Information Complexity Criterion for Cancer Classification

**DOI:** 10.1155/2015/370640

**Published:** 2015-02-19

**Authors:** Esra Pamukçu, Hamparsum Bozdogan, Sinan Çalık

**Affiliations:** ^1^Department of Statistics, Faculty of Science, Firat University, 23119 Elazig, Turkey; ^2^Department of Business Analytics and Statistics, The University of Tennessee, Knoxville, TN 37996, USA

## Abstract

Gene expression data typically are large, complex, and highly noisy. Their dimension is high with several thousand genes (i.e., features) but with only a limited number of observations (i.e., samples). Although the classical principal component analysis (PCA) method is widely used as a first standard step in dimension reduction and in supervised and unsupervised classification, it suffers from several shortcomings in the case of data sets involving undersized samples, since the sample covariance matrix degenerates and becomes singular. In this paper we address these limitations within the context of probabilistic PCA (PPCA) by introducing and developing a new and novel approach using maximum entropy covariance matrix and its hybridized smoothed covariance estimators. To reduce the dimensionality of the data and to choose the number of probabilistic PCs (PPCs) to be retained, we further introduce and develop celebrated Akaike's information criterion (AIC), consistent Akaike's information criterion (CAIC), and the information theoretic measure of complexity (ICOMP) criterion of Bozdogan. Six publicly available undersized benchmark data sets were analyzed to show the utility, flexibility, and versatility of our approach with hybridized smoothed covariance matrix estimators, which do not degenerate to perform the PPCA to reduce the dimension and to carry out supervised classification of cancer groups in high dimensions.

## 1. Introduction

The study of gene expression has been greatly facilitated by DNA microarray technology. Since DNA microarrays measure the expression of thousands of genes simultaneously, there is a great need to develop analytical methodology to analyze and to exploit the information contained in gene expression data [1, 2]. With the wealth of gene expression data from microarrays being produced, more and more new prediction, classification, and clustering techniques are being used for the analysis of the data [3]. Dimension reduction techniques such as principal component analysis (PCA) and several extended forms of PCA such as probabilistic principal component analysis (PPCA), kernel principal component analysis (KPCA) have also been proposed to analyze gene expression data. For more on these methods we refer the readers to Raychaudhuri et al. [1], Yeung and Ruzzo [2], Chen et al. [4], Yang et al. [5], Ma and Kosorok [6], and Nyamundanda et al. [7]. Although these methods are commonly used in the literature, they all inherently have their own idiosyncratic statistical difficulties in analyzing undersized samples in high dimensions due to singularity of the covariance matrix, where these difficulties have not been satisfactorily addressed in the literature. For SVM type kernel methods, although they are useful tools, they have their own limitations in the sense that they are not easily interpretable since the kernel transformation is not one-to-one and onto and the transformation is not invertible. Moreover, for a given data set the choice of the optimal kernel function and the tuning parameters in kernel-based methods has been arbitrary and has remained an unresolved academic research problem in the literature until the recent work of Liu and Bozdogan [8] and Liberati et al. [9].

The main idea of the classical PCA, for example, is to reduce the dimensionality of a data set consisting of a large number of interrelated variables, while retaining as much as possible the variation present in the data set. This is achieved by transforming the data to a new set of variables, the principal components (PCs), which are uncorrelated and ordered [10]. By applying PCA, one is implicitly assuming that the desired information is exactly provided by the percent variance explained. But such an assumption has been questioned and criticized by Scholz [11] in gene expression data analysis. Other nonexhaustive limitations of PCA can be briefly described as follows.

- When the sample size *n* is much smaller than the number of features (i.e., genes), *p*, that is, when we have *n* ≪ *p*, the maximum likelihood (ML) estimator of the covariance matrix is neither invertible nor well conditioned.
- Therefore, the classical PCA does not work well since the estimated covariance matrix becomes rank deficient. Such a case in the literature is known as* undersized sample problem* in high dimensions [12].
- PCA suffers from a probabilistic interpretation. That is, it does not have an underlying probability density model.

Estimation of the covariance matrices for small sample size and high dimensions, that is, the *n* ≪ *p* problem, is a difficult problem that has recently attracted the attention of many researchers. This problem is prevalent in* genomics*,* microarray data*,* gene sequencing, medical data mining,* and* other bioinformatics areas* as well as in* econometrics* and* predictive business modeling*. This problem is numerically one of the most challenging problems that require new and efficient computational methods.

Due to the curse of dimensionality, almost all of the classical multivariate statistical methods break down and degenerate. This means that the covariance matrix of the data cannot be computed and, as a result, one obtains only poor classification and cluster analysis results. The main reason for this problem is the high noise created by the irrelevant or redundant genes (i.e., features) present in the data.

In this paper, therefore, our main objectives are severalfold to address the limitations of the standard classic PCA and to develop and introduce a new and novel dimension reduction technique for cancer classification. These are as follows.

- To resolve the problem of small sample size and large number of dimensions, that is, the *n* ≪ *p* problem, we introduce several smoothed (or robust) covariance estimators and their hybridized forms with the neglected maximum entropy (ME) covariance matrix.
- We introduce and use probabilistic principal component analysis (PPCA) as an alternative to the classic PCA. PPCA is a probabilistic formulation of PCA based on a Gaussian latent variable model. PPCA was developed in the late 1990s and popularized by the work of Tipping and Bishop [13, 14]. PPCA is flexible and has the associated likelihood measure as the quantum of information in the data, which is needed in the model selection criteria and their computations.
- A central issue in gene expression data is the dimension reduction before any classification or clustering procedures are meaningfully applied, especially when *n* ≪ *p*. In the literature, the task of dimensionality selection has not been solved in a satisfactory way for undersized gene expression data in high dimensions. To this end, we introduce and develop celebrated Akaike's information criterion (AIC) [15], consistent Akaike's information criterion (CAIC) of Bozdogan [16], and the information theoretic measure of complexity (ICOMP) criterion of Bozdogan [17, 18] in PPCA model to choose the number of probabilistic PC components to be retained.
- Later the PPCs chosen by the information criteria are used as inputs in cancer classification using linear discriminant analysis (LDA) and quadratic discriminant analysis (QDA). The performance of these methods is compared on the well-known six benchmark gene expression data sets to emphasize the importance of the role of dimension reduction to resolve the* “curse of dimensionality”* of Bellman [19] in cancer classification problems.

Our method has distinct advantages over the previously proposed methods in that we provide analytical means of choosing the number of PPCs via the novel application of information theoretic model selection criteria to reduce the dimension automatically that can be retained and utilized in cancer classification based on sound statistical modeling procedures. We use the entire data set with its high dimensions rather than clusterwise splitting or partitioning of the variables in the analysis process due to the high dimensionality. Our approach has the generalizability property to non-Gaussian latent variable models, probabilistic independent component analysis (PICA), sparse probabilistic principal component analysis (SPPCA), and other methods. The proposed approach is efficient and computationally cost effective; the results obtained are easy to interpret in the original data space and can be used in other supervised or unsupervised cancer classification procedures.

## 2. Materials and Methods

### 2.1. Smoothed and Hybridized Covariance Estimators

#### 2.1.1. Maximum Likelihood Covariance Estimation

Let *X* be (*n* × *p*) data matrix. When the data are modeled probabilistically as Gaussian or other elliptically contoured (EC) distributions (non-Gaussians), such as Multivariate t (Mt), multivariate power exponential (MPE), multivariate Cauchy (MC), and multivariate Laplace (MLp), we must estimate the covariance matrix, Σ. When the sample size *n* is much smaller than the dimension or the number of variables (genes), *p*, the usual sample maximum likelihood (ML) estimator of Σ, given in matrix form

(1) $$\begin{array}{c} {\hat{\Sigma }}_{\text{MLE}}\equiv S=\frac{1}{n}X^{\prime }\left[I_{p}-\frac{1}{n}11^{\prime }\right]X, \end{array}$$

becomes unstable, ill-conditioned, nonpositive definite, and even singular. In (1), *X*′ denotes the transpose of *X*, *I*_*p*_ is the (*p* × *p*) identity matrix, and 1 is a column vector of one of *p*-dimensions. In such a case, we cannot compute the inverse covariance matrix or what is referred to as the* “precision matrix,”* which is needed in practically all multivariate analysis, which includes supervised and unsupervised classification and kernel-based methods, among many others. This situation is especially true in many applications where we have undersized sample problem.

The* “precision matrix,”* that is, ${\hat{\Sigma }}_{\text{MLE}}^{-1}$, depends on the determinant of ${\hat{\Sigma }}_{\text{MLE}}$ and has a bias

(2) $$\begin{array}{c} E\left(\left|{\hat{\Sigma }}_{\text{MLE}}\right|\right)=\left|\Sigma \right|\left[1-\frac{p\left(p+1\right)}{2n}+O\left(n^{-2}\right)\right] \end{array}$$

that needs to be reduced to regularize the estimated covariance matrix [20].

#### 2.1.2. Naïve Ridge Estimators of the Covariance Matrix

The usual initial resolution to singular or ill-conditioned covariance matrix problem has been the “naïve” ridge regularization

(3) $$\begin{array}{c} {\hat{\Sigma }}_{R}={\hat{\Sigma }}_{\text{ML}}+\gamma I_{p}, \end{array}$$

where *γ* > 0 is the ridge parameter and ${\hat{\Sigma }}_{R}$ indicates the ridge or regularized covariance estimator. This estimator tries to work to counteract the ill conditioned covariance by adjusting the eigenvalues of Σ. Usually, the ridge parameter, *γ*, is chosen to be very small. How large should *γ* be and how small can *γ* be have remained arbitrary and do not work well in* “large p small n”* problems.

#### 2.1.3. Smoothed Covariance Estimators

As an alternative to the “naïve” ridge regularization, many methods have been proposed to improve the estimation of the covariance matrix. All these approaches rely on the concept of shrinkage estimators and perfecting them dating back to the early work of James and Stein [21] and Stein [22] which is known as the* “Steinian type shrinkage,”* which is implicit also in many Bayesian methods as well as in the maximum entropy (ME) covariance estimation.

The idea of shrinkage estimation of the covariance matrix or what we call smoothed covariance estimators (SCEs) is to take convex combination (i.e., weighted average) of the sample estimator of Σ, $\hat{\Sigma }$, with a suitably chosen target diagonal matrix $\hat{D}$. The* shrinkage* or* smoothed estimator* of the covariance matrix then becomes a convex combination of $\hat{\Sigma }$ with some chosen target $\hat{D}$ given by

(4) $$\begin{array}{c} {\hat{\Sigma }}_{S}=\left(1-\hat{\rho }\right)\hat{\Sigma }+\hat{\rho }\hat{D}, \end{array}$$

where $\hat{\rho }$ is the optimal* shrinkage coefficient* (or* intensity*) which is a parameter between 0 and 1; that is, $0<\hat{\rho }<1$. It can be a function of the observations. The matrix $\hat{D}$ is referred to as the shrinkage target. Its naïve form can be taken to be

(5) $$\begin{array}{c} \hat{D}=\frac{\mathrm{tr}\left(\hat{\Sigma }\right)}{p}I_{p}=\left(\frac{1}{p}\overset{p}{\underset{j=1}{\sum }}\lambda_{j}\right)I_{p}=\overset{-}{\lambda }I_{p}, \end{array}$$

where tr⁡(·) denotes the trace of the matrix, *λ*_*j*_,  *j* = 1,…, *p* are the eigenvalues of the estimated sample covariance matrix, and $\overset{-}{\lambda }$ is the arithmetic mean of the eigenvalues.

The interpretation of the general form of the smoothed covariance matrix estimation in (4) is that it provides a more baseline level of variance and covariance estimation when the sample size is much smaller than the dimension of the data. By using such a weighted average, we put less weight on extremely high or low values in the estimated covariance matrix $\hat{\Sigma }$. This reduces the influence of extremely high or low values and provides a more robust and smoothed estimator. Such a structure minimizes the mean squared error (MSE); that is,

(6) $$\begin{array}{c} E\left[{\left\|\hat{\Sigma }-\Sigma \right\|}_{F}^{2}\right], \end{array}$$

where ‖·‖_*F*_^2^ denotes the squared Frobenius norm. It is difficult to compute the MSE of $\hat{\Sigma }$ without additional constraints such as the shrinkage or smoothed covariance estimator ${\hat{\Sigma }}_{S}$.

In what follows, in this paper, we introduce several robust, regularized, or smoothed covariance estimators of the form given in (4), which have been developed under several linear and quadratic loss functions.

Selected improved (or smoothed) estimates of the covariance matrix via shrinkage from Bozdogan and Howe [23] and Bozdogan [18] are as follows.

*(i) Maximum Likelihood/Empirical Bayes (MLE/EB) Covariance Estimator.* Consider

(7) $$\begin{array}{c} {\hat{\Sigma }}_{\text{MLE}/\text{EB}}={\hat{\Sigma }}_{\text{MLE}}+\frac{p-1}{n\mathrm{tr}\left({\hat{\Sigma }}_{\text{MLE}}\right)}I_{p}, \end{array}$$

where tr⁡(·) denotes the trace of the covariance matrix and *I*_*p*_ is the (*p* × *p*) identity matrix. ${\hat{\Sigma }}_{\text{MLE}/\text{EB}}$ covariance estimator was proposed by Haff [24]. When a small amount of perturbation is all that is required, ${\hat{\Sigma }}_{\text{MLE}/\text{EB}}$ has a certain appeal. It is clear that this is of the same form as the naïve ridge regularization.

*(ii) Maximum Entropy (ME) Covariance Matrix.* Consider

(8) $$\begin{array}{c} {\hat{\Sigma }}_{\text{ME}}=C+D, \end{array}$$

where *C* is the usual (nonnegative definite) dispersion matrix and *D* is a (positive definite) diagonal matrix with positive elements on the diagonal. These positive elements take the form of a weighted sum of squared differences between successive primary midpoints of the variables and these elements serve as a* ridge* in the ME covariance matrix. In other words, the construction of ME covariance matrix automatically produces the* ridge* component directly from the data without the worry of how to choose the ridge parameter as is the case in the usual ridge type of estimators. The main motivation of introducing the ME covariance matrix estimator, which has been ignored in the statistical literature, is that it makes the singular and ill-conditioned covariance matrix positive definite when we have undersized sample data such as the case in gene expression data sets. What is also interesting about the ME covariance matrix is that it uses linear and nonlinear order statistics (OS) in its computation by fully exploiting the information in the data set. The computation of the ME covariance matrix in terms of the CPU time is fast and efficient for high dimensional data and it is not heavy. A Matlab module has been written for the computation of the ME covariance matrix and utilized in our analysis in what follows.

For more on the ME covariance matrix we refer the readers to Theil and Laitinen [25], Fiebig [12], and Theil and Fiebig [26].

*(iii) Stipulated Ridge Covariance Estimator (SRE).* Consider

(9) $$\begin{array}{c} {\hat{\Sigma }}_{\text{SRE}}={\hat{\Sigma }}_{\text{MLE}}+p\left(p-1\right){\left[2n\mathrm{tr}\left({\hat{\Sigma }}_{\text{MLE}}\right)\right]}^{-1}I_{p}. \end{array}$$

We note that bias $E(|{\hat{\Sigma }}_{\text{SRE}}|)=|\Sigma |\left[1+O(n^{-2})\right]$ and $p(p-1){\left[2n\mathrm{tr}({\hat{\Sigma }}_{\text{MLE}})\right]}^{-1}=O(n^{-1})$.

*(iv) Stipulated Diagonal Covariance Estimator (SDE).* Consider

(10) $$\begin{array}{c} {\hat{\Sigma }}_{\text{SDE}}=\left(1-\hat{\rho }\right){\hat{\Sigma }}_{\text{MLE}}+\hat{\rho }\text{Diag}\left({\hat{\Sigma }}_{\text{MLE}}\right), \end{array}$$

where $\hat{\rho }=p(p-1){\left[2n(\mathrm{tr}R^{-1}-p)\right]}^{-1}$ and $R=\text{Dia}{\text{g}}^{-1/2}({\hat{\Sigma }}_{\text{MLE}}){\hat{\Sigma }}_{\text{MLE}}\text{Dia}{\text{g}}^{-1/2}({\hat{\Sigma }}_{\text{MLE}})$ is the correlation matrix. For SDE, we also note that the bias $E(|{\hat{\Sigma }}_{\text{SDE}}|)=|\Sigma |\left[1+O(n^{-2})\right]$ and $\hat{\rho }=O(n^{-1})$.

The SRE and SDE covariance estimators are due to Shurygin [20] (last student of Kolmogorov). SDE avoids scale dependence of the units of measurement of the variables. 

*(v) Convex Sum Covariance Estimator (CSE).* Preceding the series of the work of Ledoit and Wolf [27, 28], based on the quadratic loss function used by Press [29], Chen [30] proposed a convex sum covariance matrix estimator (CSE) given by

(11) $$\begin{array}{c} {\hat{\Sigma }}_{\text{CSE}}=\frac{n}{n+m}\hat{\Sigma }+\left(1-\frac{n}{n+m}\right)\hat{D}=\hat{\rho }\hat{\Sigma }+\left(1-\hat{\rho }\right)\hat{D}, \end{array}$$

where

(12) $$\begin{array}{c} \hat{D}=\frac{\mathrm{tr}\left(\hat{\Sigma }\right)}{p}I_{p}. \end{array}$$

For *p* ≥ 2 dimensions, *m* is chosen to be

(13) $$\begin{array}{c} 0<m<\frac{2\left[p\left(1+\beta \right)-2\right]}{p-\beta }, \end{array}$$

where *β* data adaptively is computed:

(14) $$\begin{array}{c} \beta =\frac{{\left(\mathrm{tr}\hat{\Sigma }\right)}^{2}}{\mathrm{tr}\left({\hat{\Sigma }}^{2}\right)}. \end{array}$$

This estimator improves upon the usual covariance by shrinking all the estimated eigenvalues toward their common mean. One obvious advantage of this estimator is that it is operational even when *n* ≪ *p*; that is, the sample size is much smaller than the dimension.

*(vi) Bozdogan's [31] Convex Sum Covariance Estimator (BCSE).* Consider

(15) $$\begin{array}{c} {\hat{\Sigma }}_{\text{BCSE}}=\hat{\rho }\hat{\Sigma }+\left(1-\hat{\rho }\right)\hat{D}, \end{array}$$

where $\hat{\rho }=1/\alpha$ and *α* is the sum of the squared deviations of each dimension and is given by

(16) $$\begin{array}{c} \alpha =\frac{1}{n-1}\overset{p}{\underset{j=1}{\sum }}\text{Var}\left(x_{j}\right). \end{array}$$

As is well known, sum of squared deviations allows the overall variability in a data set to be attributed to different types or sources of variability, with the relative importance of each being quantified by the size of each component of the overall sum of squares. We calculate the sum of squares per degree of freedom or the variance and then divide by the total degree of freedom to get (16), which is used in the estimated shrinkage target.

*(vii) Eigenvalue Stabilization of the Covariance Matrix (Thomaz [32]) (STA)*. Stabilization algorithm is as follows.

(1) Find the eigenvectors (*V*) and eigenvalues (Λ) of the covariance matrix.
(2) Compute the mean or average eigenvalue $\overset{-}{\lambda }$ of the covariance matrix:
  (17) $$\begin{array}{c} \overset{-}{\lambda }=\frac{1}{p}\overset{p}{\underset{j=1}{\sum }}\lambda_{j}=\frac{1}{p}\mathrm{tr}\left(\hat{\Sigma }\right). \end{array}$$
(3) Form a new matrix of eigenvalues based on the following largest dispersion values:
  (18) $$\begin{array}{c} \Lambda^{*}=\left[\begin{array}{cccc} \max\left(\lambda_{1},\overset{-}{\lambda }\right) & 0 & \cdots & 0\\ 0 & \ddots & \cdots & 0\\ \vdots & \vdots & \ddots & \vdots \\ 0 & 0 & \cdots & \max\left(\lambda_{p},\overset{-}{\lambda }\right) \end{array}\right]. \end{array}$$
(4) Finally, reform the modified newly stabilized covariance matrix:
  (19) $$\begin{array}{c} {\hat{\Sigma }}_{\text{STA}}=V\Lambda^{*}V. \end{array}$$

There are other smoothed covariance matrices. For space considerations, the ones above that we are studying in this paper will suffice for the results in this paper.

#### 2.1.4. Hybridized Smoothed ME Covariance Estimator

We can choose any of the smoothed covariance estimators and stabilize their eigenvalues with the STA algorithm above. However, in this paper more specifically we propose focussing our attention on the ME covariance matrix and stabilizing its eigenvalues using the eigenvalue stabilization of Thomaz [32]. Then, we hybridize our result with other smoothed covariance matrix estimators in reducing the dimension of the undersized data in high dimensions in the PPCA model using the information theoretic model selection criteria. The rationale and mathematical motivation of stabilization plus hybridization are to improve further in a straightforward way the smaller and less reliable eigenvalues of the estimated covariance matrix while trying to keep most of its larger eigenvalues unchanged before smoothing to guarantee that the eigenvalues of a nonnegative definite matrix do not become negative and to achieve positive definiteness via shrinkage. These hybrid regularized covariance estimators greatly enhance supervised and unsupervised classification error rates after the dimension reduction and for general inferences in multivariate modeling.

For example, we stabilize the ME covariance matrix and obtain

(20) $$\begin{array}{c} {\hat{\Sigma }}_{\text{ME}\_\text{STA}}=V\Lambda^{*}V. \end{array}$$

Then, we hybridize ${\hat{\Sigma }}_{\text{ME}\_\text{STA}}$, say, with the convex sum covariance estimator (CSE) and compute

(21) Σ^HCE≡Σ^ME_STA_CSE=nn+mΣ^ME_STA +(1−nn+m)tr⁡(Σ^ME_STA)pIp.

We call such a process “hybridized covariance estimator,” ${\hat{\Sigma }}_{\text{HCE}}$. Similarly, we can hybridize other smoothed covariance estimators. These hybridized smoothed (or robust) estimators of the covariance matrix overcome the singularity of the covariance matrix for undersized gene expression data sets and avoid negative eigenvalues.

**Figure 1:**
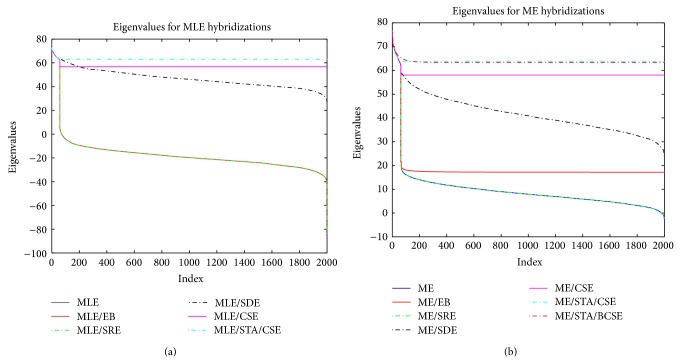
The eigenvalues for MLE and ME covariance matrices and their hybridizations with other smoothed covariance matrices.

As an illustration of our proposed approach to resolve the undersized sample problem, we discard the group structure of the colon benchmark data set for the time being and compute the usual sample covariance matrix, $\hat{\Sigma }$. Then, to remedy the singularity problem, we compute the maximum entropy (ME) covariance matrix, ${\hat{\Sigma }}_{\text{ME}}$. Later, we hybridize the ME covariance matrix with other smoothed (or robust) covariance estimators. We denote this by ${\hat{\Sigma }}_{\text{HCE}}$ as in (21). Now we compute the eigenvalues of these covariance estimators and compare them with the eigenvalues of the MLE type smoothed covariance estimator. Our results for both MLE based smoothed covariance matrices and the hybridization of the ME covariance with the smoothed covariances for the colon cancer data set are shown in Figures 1(a)-1(b).

Looking at Figure 1, we note that the eigenvalues of $\hat{\Sigma }$ are all zeros after the first eigenvalue that further shows the severe singularity since the colon data set is undersized. To remedy this problem, we can see that maximum entropy (ME) covariance matrix, ${\hat{\Sigma }}_{\text{ME}}$, recovers the singularity. ME covariance matrix estimator hybridization, ${\hat{\Sigma }}_{\text{HCE}}$, with other smoothed covariances improves the singularity further and also makes the covariance nonsingular which shows the recovery of the singularity with our approach. The corresponding eigenvalues of the final covariance estimator ${\hat{\Sigma }}_{\text{HCE}}$ are well conditioned and are positive providing a positive definite covariance matrix that can be inverted.

It is important to emphasize here that our proposed approach works for practically all the undersized benchmark gene expression data sets. It has distinct advantage over currently used methods for recovering the singularity of the estimated covariance matrices in undersized gene expression data sets or in general. It is analytical and numerically stable. It is easy to compute and efficient.

### 2.2. Information Complexity: ICOMP Criterion

In general statistical modeling and model evaluation problems, the concept of model complexity plays an important role. At the philosophical level, complexity involves notions such as connectivity patterns and the interactions of model components. Without a measure of* overall* model complexity, prediction of model behavior and assessing model quality is difficult. This requires detailed statistical analysis and computation to choose the best fitting model among a portfolio of competing models for a given finite sample [33].

The development of information theoretic measure of complexity (ICOMP) criterion has been motivated in part by Akaike's classic information criterion (AIC) given by

(22) $$\begin{array}{c} \text{AIC}\left(k\right)=-2\log L\left({\hat{\theta }}_{k}\right)+2m\left(k\right), \end{array}$$

where $L({\hat{\theta }}_{k})$ is the maximized likelihood function, ${\hat{\theta }}_{k}$ is the maximum likelihood estimate of the parameter vector *θ*_*k*_ under the model *M*_*k*_, and *m*(*k*) is the number of independent parameters estimated when *M*_*k*_ is the model and in part by* information complexity concepts* and* indices*.

Bozdogan [16] improved and extended AIC analytically in two ways without violating Akaike's principles using the established results in mathematical statistics. One of these extensions that make AIC asymptotically consistent is CAIC which is defined by

(23) $$\begin{array}{c} \text{CAIC}\left(k\right)=-2\log L\left({\hat{\theta }}_{k}\right)+m\left(k\right)\left[\log\left(n\right)+1\right]. \end{array}$$

We note that, in AIC and CAIC, the compromise takes place between the maximized log likelihood, that is, $-2\log L({\hat{\theta }}_{k})$ (the* lack-of-fit component*) and *m*(*k*), the* number of free parameters,* and *m*(*k*)[log⁡(*n*) + 1], the penalty term, where log⁡(*n*) is the natural logarithm of the sample size *n*, respectively.

In contrast to AIC and CAIC, the information complexity ICOMP criterion is based on covariance complexity index of van Emden [34]. Instead of penalizing the number of free parameters directly, ICOMP penalizes the covariance complexity of the model.

ICOMP is defined by

(24) $$\begin{array}{c} \text{ICOMP}\left(k\right)=-2\log L\left({\hat{\theta }}_{k}\right)+2C_{1}\left({\hat{\Sigma }}_{\text{Model}}\right), \end{array}$$

where ${\hat{\Sigma }}_{\text{Model}}=\;\hat{\text{Cov}}({\hat{\theta }}_{k})$ is the estimated covariance matrix of the model and $C_{1}({\hat{\Sigma }}_{\text{Model}})$ is the maximal entropic complexity given by

(25) $$\begin{array}{c} C_{1}\left({\hat{\Sigma }}_{\text{Model}}\right)=\frac{s}{2}\log\left[\frac{\mathrm{tr}{\hat{\Sigma }}_{\text{Model}}}{s}\right]-\frac{1}{2}\log\left|{\hat{\Sigma }}_{\text{Model}}\right|, \end{array}$$

where $s=\mathrm{rank}({\hat{\Sigma }}_{\text{Model}})$.

Hence, ICOMP in its idealized form is an additive composition of a term which measures the* lack of fit* (i.e.,* inference uncertainty*), a second term which measures the* complexity of the covariance matrix of the parameter estimates* of a model, which represents the* parametric uncertainty* of a model. It provides a more judicious penalty term and balances the* overfitting* and* underfitting risks* of a model compared to that of AIC. Indeed, this new approach provides an entropic general* data-adaptive penalty functional*, which is random and is an improvement over a fixed choice of penalty functional such as in AIC or its variants.

There are several forms and theoretical justifications of ICOMP. In this paper, we introduce and score only the consistent form of ICOMP, CICOMP given by

(26) CICOMP=−2log⁡L(θ^k)+k+klog⁡(n)+2C1F(Σ^Model)=CAIC+2C1F(Σ^Model).

In (26), $C_{1F}({\hat{\Sigma }}_{\text{Model}})$ represents the second order Frobenius norm characterization of the original complexity $C_{1}({\hat{\Sigma }}_{\text{Model}})$ of ${\hat{\Sigma }}_{\text{Model}}$ and in terms of eigenvalues, it is given by

(27) $$\begin{array}{c} C_{1F}\left({\hat{\Sigma }}_{\text{Model}}\right)=\frac{1}{4{{\overset{-}{\lambda }}_{a}}^{2}}\overset{s}{\underset{j=1}{\sum }}{\left(\lambda_{j}-{\overset{-}{\lambda }}_{a}\right)}^{2}, \end{array}$$

where ${\overset{-}{\lambda }}_{a}$ is the arithmetic mean of the eigenvalues of ${\hat{\Sigma }}_{\text{Model}}$.

We note that $C_{1F}({\hat{\Sigma }}_{\text{Model}})$ is* scale-invariant* and $C_{1F}({\hat{\Sigma }}_{\text{Model}})\geq 0$ with $C_{1F}({\hat{\Sigma }}_{\text{Model}})=0$ when all $\lambda_{j}={\overset{-}{\lambda }}_{a}$. Also, $C_{1F}({\hat{\Sigma }}_{\text{Model}})$ measures the* relative variation in the eigenvalues* rather than* absolute variation of the eigenvalues*. For more details on the analytical developments of these information complexity criteria, we will refer the readers to Bozdogan [16–18, 23, 31, 33, 35–40].

A model with the minimum information criteria score is chosen to be the best model among the competing alternative models.

When *n* ≪ *p*, in the next section, we introduce and develop a novel approach to reduce the dimension of large microarray data sets for supervised and unsupervised classification. Although the use of the classic principal component analysis (PCA) has been commonly used method for dimension reduction, it is problematic especially in gene expression data analysis since the data sets are extremely undersized and high dimensional. The eigenvalue *λ*_*j*_ is not a good estimator of the variance of the *j*th PC, since the estimated covariance matrix $\hat{\Sigma }$ is singular with *p* − *n* + 1 degenerate zero eigenvalues. In this sense, in the literature the task of dimensionality selection has not been solved in a satisfactory way for undersized samples.

### 2.3. Dimension Reduction with Probabilistic Principal Component Analysis (PPCA)

#### 2.3.1. Gaussian Latent Variable Model

Probabilistic principal component analysis (PPCA) is a Gaussian probabilistic generalization of PCA. It has been used in many areas. In its formulation PPCA presumes a linear latent variable model relating an observed variable with a latent variable that is inferred only from observed variable through a linear mapping called factor loading. PPCA offers several advantages over the PCA. These include hybridized regularization procedures such as the one proposed in this paper, model selection for dimension reduction, easy interpretation of the results, and its generalizability to other distributional models other than the Gaussian model. PPCA can also be viewed as a marginal density or a predictive model in its setup.

In this paper, we use the maximum likelihood estimates (MLEs) of the parameters of PPCA. The MLE approach computationally is efficient and works well for high dimensional data.

To be more specific, following Tipping and Bishop [13, 14] in matrix notation, we express the probabilistic principal component analysis (PPCA) model as a mapping (or transformation) from latent space into the data space via

(28) $$\begin{array}{c} x=\Lambda f+\mu +\varepsilon , \end{array}$$

where *x* is a (*p* × 1) vector of high dimensional observed variables (genes), Λ is a (*p* × *m*) factor loading matrix that represents a linear transformation, that is, Λ : *f* → *x*, *f* is (*m* × 1) latent variable, *μ* is a (*p* × 1) mean vector, and *ε* is (*p* × 1) multivariate Gaussian random error (or noise) for *x* independent of the latent variable *f*.

We note that the latent variable model in (28) clearly shows the idea of dimensionality reduction since a high dimensional observation vector *x* can be represented by a low-dimensional latent variable *f* through the mapping Λ such that *m* ≤ *p*, where *m* is the number of latent variables (PPCs) and *p* is the dimension of the data.

In order to be able to introduce the probabilistic modeling of *x*, we assume that

- the probability density of *f* is a unit spherical Gaussian: *f* ~ *N*(0, *I*_*m*_),
- the probability density of *ε* is spherical Gaussian: *ε* ~ *N*(0, Ψ) = *N*(0, *σ* ^2^ *I*_*p*_).

Finally, the observed variable *x* ends up with a Gaussian probability model

(29) $$\begin{array}{c} x\sim N\left(\mu ,\Lambda \Lambda^{\prime }+\sigma^{2}I_{p}\right), \end{array}$$

where the (*p* × *p*) covariance matrix of the observation vector *x* is

(30) $$\begin{array}{c} \text{Cov}\left(x\right)\equiv \Sigma =\Lambda \Lambda^{\prime }+\sigma^{2}I_{p}. \end{array}$$

#### 2.3.2. Probability Model

The probability distribution *p*(*x*∣*f*) is formulated with the help of the probability model of the random error *ε* given by

(31) $$\begin{array}{c} p\left(\varepsilon ;\sigma^{2}\right)={\left(2\pi \sigma^{2}\right)}^{-p/2}\exp\left(-\frac{1}{2}\varepsilon^{\prime }\varepsilon \right). \end{array}$$

Since *ε* = *x* − Λ*f* − *μ*, the conditional probability of *x* given *f*, that is, *p*(*x*∣*f*), can be obtained from *p*(*ε*). This is given by

(32) $$\begin{array}{c} p\left(x\;|\;f;\Lambda ,\mu ,\sigma^{2}\right)={\left(2\pi \sigma^{2}\right)}^{-p/2}\exp\left(-\frac{1}{2}{\left\|x-\Lambda f-\mu \right\|}^{2}\right), \end{array}$$

where ‖·‖^2^ denotes the square of the matrix norm.

Under the Gaussian prior probability the distribution of *f* is given by

(33) $$\begin{array}{c} p\left(f\right)={\left(2\pi \right)}^{-m/2}\exp\left(-\frac{1}{2}f^{\prime }f\right). \end{array}$$

Since *f* ~ *N*(0, *I*_*m*_), the marginal probability distribution, *p*(*x*) is

(34) px≡p(x;Λ,μ,σ2)=∫fpx ∣ fp(f)df=2π−p/2Σ−1/2exp⁡−12x−μ′Σ−1(x−μ)=Nx ∣ μ,ΛΛ′+σ2Ip,

which is again a Gaussian model with covariance matrix

(35) $$\begin{array}{c} \text{Cov}\left(x\right)\equiv \Sigma =\Lambda \Lambda^{\prime }+\sigma^{2}I_{p}. \end{array}$$

In addition, using the Bayes rule, we can also directly obtain the posterior probability distribution of *f* given *x*; that is, we can obtain *p*(*f*∣*x*)_Post_ such that

(36) $$\begin{array}{c} p{\left(f\;|\;x\right)}_{\text{Post}}\sim N_{m}\left(M^{-1}\Lambda^{\prime }\left(x-\mu \right),\sigma^{-2}M\right), \end{array}$$

where an (*m* × *m*) matrix *M* is given by *M* = ΛΛ′ + *σ* ^2^ *I*_*m*_. We note that the posterior mean of the latent variable *f* depends on the observation vector *x*, whereas the posterior covariance matrix *M* is independent of *x*.

From the above setup, we observe that PPCA is a constrained covariance model, since *M* is (*m* × *m*) while Σ is (*p* × *p*).

#### 2.3.3. Maximum Likelihood Estimates of the Parameters

The goal of PPCA is to estimate the unknown parameters Λ, *μ* and the noise variance *σ* ^2^ from *n* observations *x* = (*x*_1_, *x*_2_,…, *x*_*n*_) using the method of maximum likelihood. To achieve this, we need to produce the likelihood and log likelihood function of the model. The likelihood function for a given *n* observations is given by

(37) $$\begin{array}{c} L\left(\Lambda ,\mu ,\sigma^{2}\;|\;x\right)=\overset{n}{\underset{i=1}{\prod }}p(x_{i};\Lambda ,\mu ,\sigma^{2}). \end{array}$$

Thus,

(38) LΛ,μ,σ2 ∣ x =2π−np/2Σ−n/2exp⁡−12∑i=1nxi−μ′Σ−1(xi−μ) =2π−np/2Σ−n/2exp⁡−12tr⁡Σ−1S,

where

(39) $$\begin{array}{c} S=\frac{1}{n}\overset{n}{\underset{i=1}{\sum }}\left(x_{i}-\hat{\mu }\right){\left(x_{i}-\hat{\mu }\right)}^{\prime }\; \end{array}$$

is the sample covariance matrix of the observed data and $\hat{\mu }$ is the maximum likelihood estimate of the mean vector *μ*, which is given by

(40) $$\begin{array}{c} \hat{\mu }=\frac{1}{n}\overset{n}{\underset{i=1}{\sum }}x_{i}=\overset{-}{x} \end{array}$$

regardless of Λ and *σ* ^2^.

The log likelihood function is, therefore, given by

(41) log⁡LΛ,μ,σ2 ∣ x=−np2log⁡(2π)−n2log⁡Σ −n2tr⁡Σ−1S.

Maximization with respect to Λ and *σ* ^2^ is more complex but nevertheless has an exact closed form solution. As shown in Tipping and Bishop [13, 14], without going into details, explicit maximum likelihood estimates of Λ and *σ* ^2^ are obtained from (30) given by

(42) $$\begin{array}{c} {\hat{\Lambda }}_{\text{ML}}=U_{m}{\left(L_{m}-\sigma^{2}I_{m}\right)}^{1/2}R, \end{array}$$

(43) $$\begin{array}{c} {{\hat{\sigma }}^{2}}_{\text{ML}}=\frac{1}{p-m}\overset{p}{\underset{j=m+1}{\sum }}\lambda_{j}. \end{array}$$

In (42), *U*_*m*_ is a (*p* × *m*) matrix whose columns are given by the leading eigenvectors (PCs) of the sample covariance matrix *S*, the (*m* × *m*) diagonal matrix *L*_*m*_ has elements given by the corresponding eigenvalues *λ*_*j*_, and *R* is an arbitrary orthogonal matrix. For convenience often *R* is chosen to be the identity matrix; that is, *R* = *I*. When *R* = *I*, we note that the columns of $\hat{\Lambda }$ are the PCs scaled by variance parameter *λ*_*j*_ − *σ* ^2^. The maximum likelihood estimator, ${\hat{\sigma }}^{2}$, of the noise variance is nothing but the average of the left-out eigenvalues of the sample covariance matrix *S* given in (39).

Assuming that $\hat{\Lambda }$ has *m* ^*^ ≤ *m* nonzero eigenvalues (or singular values) and substituting

(44) $$\begin{array}{c} \hat{\Lambda }=U_{m^{*}}{\left(L_{m^{*}}-\sigma^{2}I_{m^{*}}\right)}^{1/2}R \end{array}$$

into the log likelihood function, we have

(45) log⁡LΛ^,μ,σ2=−np2log⁡2π−n2∑j=1m∗log⁡λj −n21σ2∑j=m∗+1pλj−n2(p−m∗)log⁡(σ2) −n2(m∗).

Maximizing (45) with respect to *σ* ^2^ gives

(46) $$\begin{array}{c} {\hat{\sigma }}^{2}=\frac{1}{p-m^{*}}\overset{p}{\underset{j=m^{*}+1}{\sum }}\lambda_{j}. \end{array}$$

After some work and simplifications, the maximized log likelihood function is given by

(47) log⁡LΛ^,μ^,σ^2=−np2log⁡2π−n2∑j=1m∗log⁡λj −np−m∗2log⁡(σ^2)−np2,

where the second term is the sum of the log of the eigenvalues corresponding to the included variables. At these parameter values, the estimated covariance is $\hat{\text{Cov}}(x)=U_{p}\hat{L}U_{p}^{\prime }$, where *U*_*p*_ contains all the eigenvalues of $\hat{\Sigma }$. $\hat{L}$ is almost a (*p* × *p*) matrix with eigenvalues of $\hat{\Sigma }$ on the diagonals given by

(48) $$\begin{array}{c} \hat{L}=\left[\begin{array}{cccc} \lambda_{1}^{*} & & & 0\\ & \lambda_{1}^{*} & & \\ & & \ddots & \\ 0 & & & \lambda_{p}^{*} \end{array}\right],\;\text{for}\;\lambda_{j}^{*}=\left\{\begin{array}{cc} \lambda_{j}^{*} & \text{if}\;I\left(j\right)=1\\ {\hat{\sigma }}^{2} & \text{otherwise}, \end{array}\right. \end{array}$$

where *I*(*j*) is an indicator function [40].

Minus twice the maximized log likelihood is

(49) −2log⁡LΛ^,μ^,σ^2=nplog⁡(2π)+n∑j=1m∗log⁡(λj) +n(p−m∗)log⁡(σ^2)+np.

This gives us the lack-of-fit component in the information criteria, which we need in deriving them. In (49), the first term and the last term do not involve *m* ^*^, the number of nonzero eigenvalues (or singular values), so they will not affect the comparison of the models and can be dropped. Hence, approximate maximized log likelihood becomes

(50) $$\begin{array}{c} -2\log L^{*}\left(\hat{\Lambda },\hat{\mu },{\hat{\sigma }}^{2}\right)=n\overset{m^{*}}{\underset{j=1}{\sum }}\log\left(\lambda_{j}\right)+n\left(p-m^{*}\right)\log\left({\hat{\sigma }}^{2}\right). \end{array}$$

In addition to the maximum likelihood estimation, there is also the Expectation and Maximization (EM) algorithm of Dempster and Laird [41] to obtain the MLEs of the parameters of the PPCA model. However, our experience is that EM algorithm is too slow to converge in small sample and high dimensional data sets without the use of some smoothing methods.

#### 2.3.4. Choosing the Number of PPCs: Derived Forms of the Information Criteria

How many eigenvalues or eigenvectors are needed in the probabilistic PCA (PPCA) model? To answer this question, we now show the derived forms of several information based model selection criteria to choose number of eigenvalues needed in the PPCA model. These criteria are computed using the hybridized smoothed covariance matrix of the original data as we discussed above. Smoothed eigenvalues and eigenvectors are sorted and *k*_max⁡_ = min⁡(*p* − 1, *n* − 2) heuristics is used to extract the maximum number of PPCs. The approximate computational derived forms of the information criteria are given as follows.

First, we give Akaike's information criterion (AIC):

(51) AICk=−2log⁡L∗(Λ^,μ^,σ^2)+2k=n∑j=1m∗log⁡(λj)+n(p−m∗)log⁡(σ^2) +2k,

where *k* = *m* ^*^ *p* + 1 − *m* ^*^(*m* ^*^ − 1)/2 is the number of free parameters estimated in the model.

Next we give the approximate computational forms of Bozdogan's [16, 31] consistent AIC (CAIC) and consistent ICOMP (CICOMP) criteria. Consider

(52) CAICk=−2log⁡L∗(Λ^,μ^,σ^2)+k[log⁡(n)+1]=n∑j=1m∗log⁡⁡(λj)+n(p−m∗)log⁡⁡(σ^2) +k[log⁡(n)+1],CICOMP=n∑j=1m∗log⁡⁡λj+n(p−m∗)log⁡⁡(σ^2)   +k[log⁡n+1]+2C1F(Σ^HCE)=CAIC(k)+2C1F(Σ^HCE),

where *C*_1*F*_(·) is the Frobenius norm characterization of the entropic complexity measure of ${\hat{\Sigma }}_{\text{HYB}}$, which is given by

(53) C1FΣ^HCE=1str⁡Σ^HCE′Σ^HCE−tr⁡Σ^HCEs2=14λ−2∑j=1sλj−λ−2,

where $s=\mathrm{rank}({\hat{\Sigma }}_{\text{HCE}})$ and *λ*_*j*_ is *j*th eigenvalue of ${\hat{\Sigma }}_{\text{HCE}}$, the hybridized smoothed covariance matrix, and $\overset{-}{\lambda }$ is the average of the eigenvalues.

We use these criteria to choose the number of PPCs in the data to reduce the dimension. As noted the manifestation of the singular covariance matrices has been resolved by using the new hybridized smoothed (or robust) estimators of the covariance matrix when the sample size for the gene expression data is much smaller than the number of dimensions. The minimum of the criteria is chosen to be the best approximating dimension.

## 3. Numerical Examples Based on Benchmark Gene Expression Data Sets

To study the effectiveness, versatility, and the utility of our proposed method, in this section we report the results of our analysis on six publicly available benchmark gene expression data sets. Although these benchmark data sets are relatively old microarray gene expression data sets, our methodology is useful for the analysis of high quality of genomic data obtained from next generation sequencing (NGS) technologies as well.

We compare our results with the currently available findings on the same data sets using other high dimensional classification techniques. The list of the benchmark data sets we considered is represented in Table 1.

We note that all these six data sets are extremely undersized with high dimensions with two (cancerous tumor and normal groups), three (subtypes of lymphoma), four (with different tumor types), and five groups (with different tumor types). Further, group sample sizes are also extremely undersized leading to the manifestation of singular covariance matrices.

### 3.1. Supervised Classification Using LDA and QDA: A Motivational Example

In order to motivate the difficulty of the supervised classification of these benchmark data sets, we use first 5, 10, and 15 genes to carry out the LDA, QDA to classify the observations.  Table 2 summarizes the results obtained from both LDA and QDA.

Looking at Table 2, we see high percentage of misclassification rates across different benchmark data sets. In the analysis, we cannot go beyond 15 original genes to analyze these data sets since the estimated class covariance matrices become notoriously singular and any further results obtained from these classification procedures become not reliable and potentially misleading. What this means is that we cannot capture the variability, the structure, and the full information in these data sets. This is crucial in treatment and prognosis of classification of cancerous tumors in the early face of discovery. Therefore, a word of caution is that one should not haphazardly utilize these supervised classification procedures automatically when we have undersized sample with high dimensions to carry out the usual discriminant analysis between groups of samples.

### 3.2. Dimension Reduction and Supervised Classification Using PPCA and Information Criteria

Next, we carried out PPCA using AIC, CAIC, and consistent ICOMP (CICOMP) criterion using the hybridized smoothed (or robust) covariance estimator. We used several combinations of hybridization. Contrary to the claimed results, just using the smoothed covariance estimators alone in undersized samples, it is not fully guaranteed to get all positive eigenvalues to make the estimated covariance matrix become positive definite and well-conditioned. It is because of this that we considered all the combinations of the hybridized covariance matrices and chose those hybrid smoothed covariance estimators to reduce the dimension of the PPCA model. We interpret PPCA results as density estimation that operates exclusively on the eigenvalues of the hybridized smoothed covariance matrix.

After the dimension reduction using PPCA, the final stage of our analysis consists of classification using the newly transformed PPCA data.

**Figure 2:**
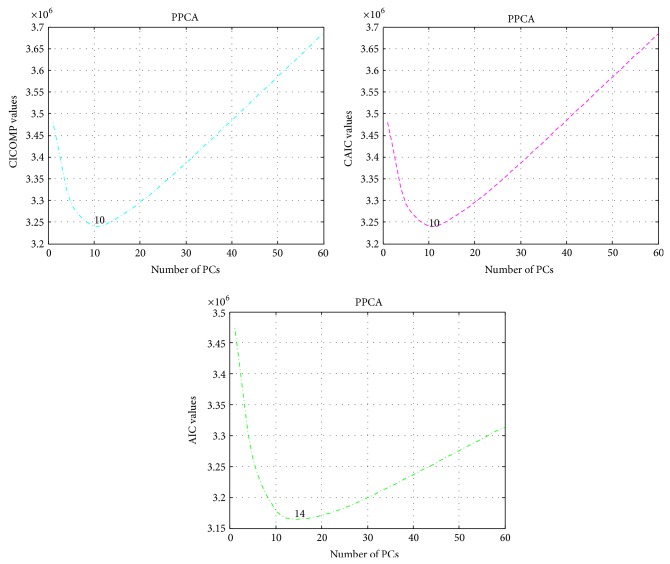
The plots of the minimum values of three information criteria in choosing the number of best PPCs for colon data set.

As an illustration, Figure 2 shows the plots of the minimum values of three information criteria in choosing the number of best PPCs for the colon data set.

After reducing the dimension of all the benchmark gene expression data sets, the results from PPCA dimension reduction and classification using newly transformed PPCA data for AIC, CAIC, and ICOMP solutions are summarized in Tables 3 and 4.

Looking at Tables 3 and 4, we see the remarkable performance of the LDA and QDA classifiers with our approach using PPCA in terms of the misclassification error rates after the dimension reduction. Although the performances vary across different data sets, which is expected, our results are encouraging since the PPCs as latent variables are the linear combinations of all the original genes, and we can use these transformed data as our new data in our subsequent analysis without losing much information in the original data sets. The other important point to mention here is that we have not altered or perturbed the original data with our approach.

Based on these results, we observe that, using the combination of PPCA and the usual classification methods with the proposed new approach, we do not overfit the model as the case is for many supervised learning methods in gene expression data analysis.

For example, if we compare our results on the same six benchmark data sets with that of the classification results obtained by Dettling [42] using seven classification techniques,* Bagboost*,* Boosting*,* Random Forest*,* SVM*,* PAM*,* DLDA*, and* kNN* classifiers, our percent misclassification error rates are much better across the six data sets using the LDA and QDA classifier. For example, for Leukemia data set Dettling's best result with SVM gives 1.83% misclassification error rate, with our approach PPCA + LDA giving 1.38% and PPCA + QDA giving 0.0% misclassification error rate. For colon data, PPCA + QDA gives 14.52% misclassification error rate as compared to* Random Forest* method, which is 14.86%. For prostate data set, with PPCA + QDA, we obtain 7.84% misclassification error rate as compared to the best result with* Bagboost*, which is 7.53%, and with SVM 7.88%. For lymphoma data set, with PPCA + QDA we get 0.0% misclassification error rate, and Dettling gets 1.62% with* Random Forest* 1.24% and with* Bagboost* 1.62% misclassification error rates. For the SRBCT data set the best result is with* Bagboost* 1.24% as opposed to our result with PPCA + LDA and QDA; it is 0.0%. Finally for the brain data none of the seven classification techniques used by Dettling gives good error rates. These error rates are all above 20% which is quite high. With our approach, our results with PPCA + LDA give 9.52% and with PPCA + QDA give 4.76% misclassification error rates. Further, we do not need to use the usual PCA on the estimated covariance matrix of the pooled samples and rotate the data and then carry out existing high dimensional classifiers.

## 4. Conclusions, Discussion, and Future Work

In this paper we introduced a general novel and new method to resolve the inherent problems in undersized gene expression data via the hybridized smoothed covariance estimators to guarantee positive definiteness of the estimated covariance matrix via hybridization with smoothed covariance estimators in undersized samples with high dimensions. We showed on six benchmark data sets how to reduce the dimension using three information theoretic model selection criteria to drive and study the cancer tumor classification problem using the hybridized covariance estimators. Our results are unique in the sense that if we use the original data sets using the usual covariance estimator, there are singularities in the class covariance matrices according to QDA results. What this means is that the conventional multivariate techniques such as classical PCA to reduce the dimensionality in gene expression data sets do not work and they degenerate since the covariance matrices become singular. This point has been overlooked in the statistical literature. To our best knowledge, there does not exist a new and novel method to make the estimated covariance matrix become positive definite that can be inverted for the original data and guarantee always-positive eigenvalues.

Although we analyzed and demonstrated our results on several publicly available benchmark relatively old microarray gene expression data sets, our novel methodology is useful for the analysis of high quality of genomic data obtained from next generation sequencing (NGS) technologies. As is well known, NGS technologies opened the floodgates for quality new genomic data. NGS instruments, the so-called second-generation sequencers, generate large volumes of data compared with conventional Sanger sequencers. There is a pressing need for new and novel methods such as the ones presented in this paper to analyze and interpret genomic data better with undersized samples and high dimensions. For example, the identification of new disease genes may provide new therapeutic targets and improve the predictive abilities of genetic testing. This will help clinical sequencing of patients suffering from disease and may eventually guide diagnosis and treatment decisions in personalized medicine.

Our proposed method can be used to solve new problems and challenges present in the analysis of NGS data in bioinformatics and other biomedical applications.

The use and introduction of the information criteria may be new for dimension reduction in PPCA model, but our approach is confined to dimension reduction. It has many other applications in predictive computational modeling of physical and biological diverse materials also using machine-learning methods in choosing the optimal kernel function among competing alternative kernels. For more on applications of these, see, for example, Liu and Bozdogan [8] and Liberati et al. [9].

In the literature, often support vector type kernelization is used, but SVM is not free from ill conditioning. In the SVM framework, reduced-rank approximations have been used to carry out the analysis in the reproducing kernel Hilbert feature space (RKHS). With our approach, it is now possible to handle singular ill-posed problems in the analysis of gene expression and NGS data.

In our future study, we will extend the results of this work to cover non-Gaussian PPCA, kernel density PPCA, probabilistic independent component analysis (PICA), and unsupervised* mixture model cluster analysis* problems as well as choosing the best subset of the genes using the genetic algorithm (GA) with ICOMP as the fitness function and compare their performances with other strategies. Our results will be published and reported separately.

*Availability of Supporting Data*. The publicly data sets are available at http://www.biomedcentral.com/1471-2105/7/228#B9.

## Figures and Tables

**Table 1:** Benchmark gene expression data sets.

Data set	Reference	*n*	*p*	Number of groups	Definition of groups
Leukemia	Golub et al. [43]	72	3571	2	Subtypes of leukemia
Colon	Alon et al. [44]	62	2000	2	Tumor/normal tissue
Prostate	Singh et al. [45]	102	6033	2	Tumor/normal tissue
Lymphoma	Alizadeh et al. [46]	62	4026	3	Subtypes of lymphoma
SRBCT	Khan et al. [47]	63	2308	4	Different tumor types
Brain	Pomeroy et al. [48]	42	5597	5	Different tumor types

**Table 2:** Classification results of benchmark gene expression data sets using the 5, 10, and 15 original genes.

Data sets	Original number of dimensions	LDA misclassification error rates			QDA misclassification error rates		
		5 genes	10 genes	15 genes	5 genes	10 genes	15 genes
Leukemia	3571	43.1%	33.3%	20.1%	31.9%	22.2%	6.9%
Colon	2000	35.4%	32.2%	25.8%	32.2%	16.1%	16.1%
Prostate	6033	34.3%	36.27%	18.6%	30.4%	25.5%	18.6%
Lymphoma	4026	30.6%	29.0%	16.1%	19.4%	1.61%^*^	3.22%^*^
SRBCT	2308	31.8%	12.7%	17.5%	15.9%	3.18%^*^	0.0%^*^
Brain	5597	45.2%	30.9%	14.2%	42.8%^*^	9.5%^*^	16.6%^*^

^*^There are singularities in the covariance matrices.

**Table 3:** Results from the dimension reduction with PPCA + classification of benchmark data sets using AIC.

Data sets	Original number of dimensions	Hybrid smoothed Covs	Dimension reduction	LDA misclassification error rates	QDA misclassification error rates
Leukemia	3571	ME/STA/BCSE	6	15.28%	26.3%
Colon	2000	ME/STA/CSE	14	19.35%	12.9%
Prostate	6033	ME/STA/BCSE	6	36.27%	34.31%
Lymphoma	4026	ME/STA/BCSE	15	4.83%	1.61%
SRBCT	2308	ME/STA/BCSE	15	9.52%	0.0%
Brain	5597	ME/STA/CSE	14	2.38%	4.76%

**Table 4:** Results from the dimension reduction with PPCA + classification of benchmark data sets using CAIC and CICOMP.

Data sets	Original number of dimensions	Hybrid smoothed Covs	Dimension reduction	LDA misclassification error rates	QDA misclassification error rates
Leukemia	3571	ME/STA/CSE	8	1.38%	0.0%
Colon	2000	ME/STA/CSE	10	22.58%	14.52%
Prostate	6033	ME/STA/CSE	12	3.92%	7.84%
Lymphoma	4026	ME/STA/CSE	8	0.0%	0.0%
SRBCT	2308	ME/STA/CSE	8	0.0%	0.0%
Brain	5597	ME/STA/CSE	4	9.52%	4.76%
